# An Optical Fiber Sensor Based on La_2_O_2_S:Eu Scintillator for Detecting Ultraviolet Radiation in Real-Time

**DOI:** 10.3390/s18113754

**Published:** 2018-11-02

**Authors:** Yongji Yan, Xu Zhang, Haopeng Li, Yu Ma, Tianci Xie, Zhuang Qin, Shuangqiang Liu, Weimin Sun, Elfed Lewis

**Affiliations:** 1 Key Lab of In-fiber Integrated Optics, Ministry Education of China, Harbin Engineering University, Harbin 150001, China; yongjiyan@hrbeu.edu.cn (Y.Y.); zhangxu_@hrbeu.edu.cn (X.Z.); 014116307@hrbeu.edu.cn (H.L.); vicky_mic@hrbeu.edu.cn (Y.M.); yuci@hrbeu.edu.cn (T.X.); qinzhuang13@163.com (Z.Q.); liukunwu@hrbeu.edu.cn (S.L.); 2 Optical Fiber Sensors Research Centre, University of Limerick, Castletroy, Limerick, Ireland; Elfed.Lewis@ul.ie

**Keywords:** ultraviolet optical fiber sensors, UV sensors, La_2_O_2_S:Eu scintillator, real-time UV sensors

## Abstract

A novel ultraviolet (UV) optical fiber sensor (UVOFS) based on the scintillating material La_2_O_2_S:Eu has been designed, tested, and its performance compared with other scintillating materials and other conventional UV detectors. The UVOFS is based on PMMA (polymethyl methacrylate) optical fiber which includes a scintillating material. Scintillating materials provide a unique opportunity to measure UV light intensity even in the presence of strong electromagnetic interference. Five scintillating materials were compared in order to select the most appropriate one for the UVOFS. The characteristics of the sensor are reported, including a highly linear response to radiation intensity, reproducibility, temperature response, and response time (to pulsed light) based on emission from a UV source (UV fluorescence tube) centered on a wavelength of 308 nm. A direct comparison with the commercially available semiconductor-based UV sensor proves the UVOFS of this investigation shows superior performance in terms of accuracy, long-term reliability, response time and linearity.

## 1. Introduction

Ultraviolet light (UV) is a general term for radiation in the wavelength range from 10 to 400 nm in the electromagnetic spectrum and cannot stimulate anthropic vision. Depending on the wavelength, the ultraviolet spectral region is generally divided into three bands, long-wavelength ultraviolet UV-A (320–400 nm), medium-wavelength ultraviolet UV-B (270–320 nm), and short-wavelength ultraviolet UV-C (200–270 nm) [1]. A small amount of UV radiation is beneficial to the human body, but if exposed to ultraviolet light in the range 280–380 nm for a long time, the risk of skin cancer [2] is greatly increased and the retina can also be damaged [3,4]. However, due to the unique properties of UV, it has penetrated widespread industrial, medical, and other applications [5]. For example, ultraviolet germicidal irradiation (200–280 nm) is commonly used to neutralize bacteria and viruses as it has a high sterilization efficiency, no secondary pollution, and is considered safe and reliable [6]. High-voltage equipment can produce an electrical discharge which in turn produces UV light in the wavelength range λ = 230–280 nm. A UV sensor operating only within the 200–280 nm window can accurately and effectively measure the discharge point of high-voltage equipment by removing the complex noise caused by ambient light variations [7]. According to the Rayleigh scattering law, UV in the range of 200–280 nm can also be used for UV communication for military applications due to its strong scattering properties and the resulting limited propagation area caused by strong absorption in the atmosphere [8]. In such circumstances, measurement of UV radiation intensity is an important issue that demands rapid improvement of detector technology.

**Figure 1:**
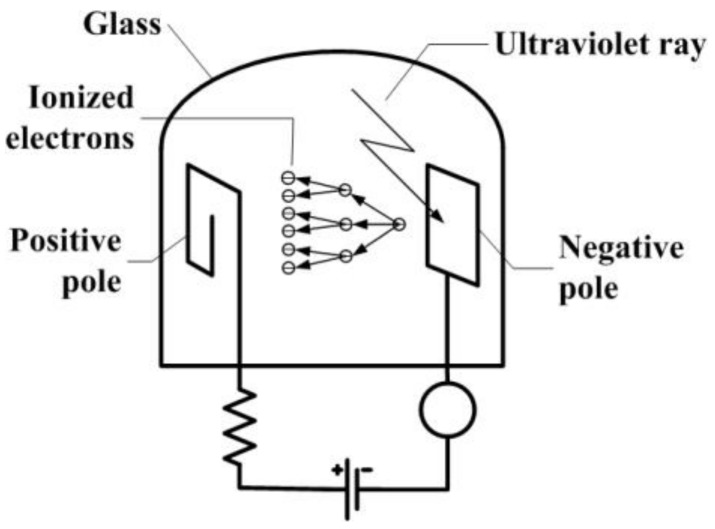
The photosensitive tube schematic diagram.

To measure UV radiation, there are currently two classes of measuring devices, namely ultraviolet photosensitive tubes and semiconductor detectors. Figure 1 shows the schematic and operating principle of UV photosensitive tubes based on the theory of successive electron avalanches. With the help of a peripheral circuit, this device outputs pulse signals whose frequency increases with increasing UV irradiation intensity. The second group of UV detectors consist of semiconductor devices whose operating principle is based on the internal photoelectric effect. These semiconductors can detect optical signals which also extend into the visible range. The photosensitive tube has some disadvantages compared to semiconductor devices such as bulk, fragility, cost, etc. With the advantages of low cost and small volume (low power), the semiconductor UV detector is more convenient to use and maintain than the photosensitive tube, but the sensitivity is lower. Additionally, both classes of UV detectors referred to above are not immune to electromagnetic interference, meaning that they cannot be used in any environment in which an electromagnetic disturbance occurs, e.g., in proximity to high voltage devices [1] as well as in the presence of strong magnetic fields.

The well-known phenomenon of scintillation can be used to overcome this problem of being susceptible to electromagnetic interference [9]. Moreover, scintillating optical fiber sensors have many advantages, such as having a miniature structure, being lightweight, having a high sensitivity, etc., [9,10,11,12]. In addition, they can be used in water. Some UV sensors based on optical fibers coated with phosphor doped polymers were reported in recent decades [13,14]. McSherry et al. applied an optical fiber sensor coated with a mixture of phosphor and epoxy to detect UV radiation at 254 nm [15], the peak Germicidal wavelength value. The use of a coated fluorescent material structure and geometrical considerations often results in the optical signal coupling efficiency being low. Miluski et al. used the efficient luminescence of a polymethyl methacrylate (PMMA) matrix doped with Europium to fabricate an optical sensor for the detection of UV radiation [16]. In both cases, the sensors encountered problems with poor linearity in the measured range.

In this paper five scintillating materials which absorb UV light and emit visible fluorescence light were compared. Based on the results of this comparison, the material La_2_O_2_S:Eu was selected as the optimum for use in the ultraviolet optical fiber sensor (UVOFS) of this investigation. In this evaluation, the sensor characteristics assessed included linearity, repeatability, temperature response, and time response (sampling frequency).

## 2. Design and Principle of UVOFS

### 2.1. Structure of the UVOFS

**Figure 2:**
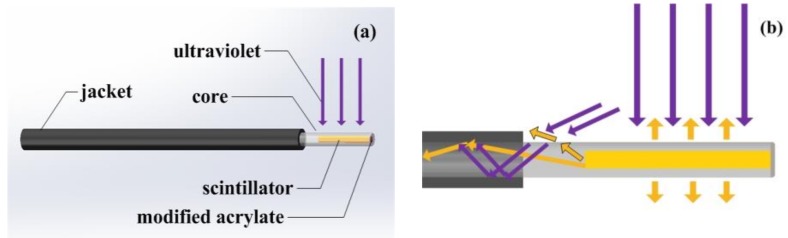
The structure of the UVOFS. (**a**) the geometry of the UVOFS, (**b**) the schematic of the optical signal transmission in the sensor’s probe.

Figure 2 shows the concept of the operation and structure of the UVOFS of this investigation. The sensor probe is a 1 mm core diameter PMMA fiber (Eska SH-4001, Minato-ku, Tokyo, Japan) with a 10 mm length of its jacket removed at the sensing end of the fiber. A small hole was drilled at the end face of the fiber within a core of a 0.5 mm diameter and 5 mm depth. The small hole was tightly filled with a scintillating material and glued with modified acrylate (AIBIDA AB glue).

**Figure 3:**
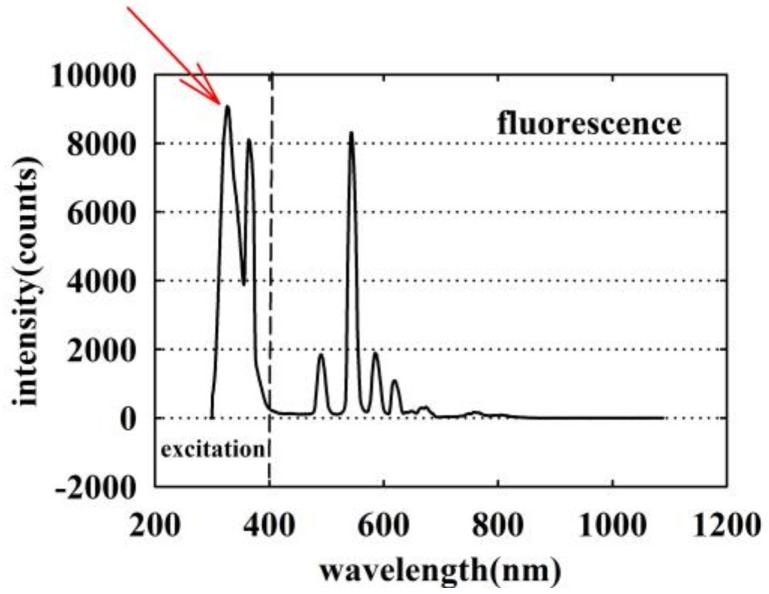
The experimentally measured optical spectrum of the La_2_O_2_S:Tb scintillator based sensor.

When UV light illuminates the exposed end of the fiber, the scintillating material in the PMMA core interacts with the UV light and emits visible fluorescence light as shown in Figure 2. A part of the visible fluorescence light propagates along the PMMA fiber and is detected using a distal fluorescence spectrometer (Ocean Optics QEPRO QEP00331, Seminole, FL, USA). Some obliquely incident UV light also couples into the fiber as shown in Figure 2b. It is possible to use part of the UV light that is transmitted in the fiber to monitor the UV radiation intensity received by the sensor. Figure 3 shows the spectrum of the La_2_O_2_S:Tb scintillator based sensor of Figure 2 captured experimentally using the optical spectrum analyzer based system described in further detail in the next section. The part of the graph to the left of the dashed line represents the UV light coupled into the fiber, and to the right side is the fluorescent signal emitted by the sensitive material which was also measured at the end of the fiber. The maximum value of the main peak in Figure 3 is read as the UV intensity and is indicated by the red arrow on Figure 3.

### 2.2. Scintillation Material Emission Principle

The scintillating material can effectively absorb high-energy rays or high-energy particles and emit UV or visible light. Scintillating materials play an important role in high-energy physics, medical diagnoses, and industrial non-destructive detection.

Five scintillating materials were investigated as possible candidates for the UVOFS. The CsI:Tl crystal has a high light output and an emission wavelength of about 560 nm. It is a commonly used material for high energy radiation detection, e.g., X-Rays and Gamma Rays. Gd_2_O_2_S is an important rare earth oxysulfide with a hexagonal structure and a wide band gap (4.2 to 4.8 eV). Among the different variations on this material, Tb^3+^ doped Gd_2_O_2_S has a high absorption efficiency and excellent conversion efficiency to visible light. The Pr^3+^ doped Gd_2_O_2_S scintillator powder has the advantages of short decay time coupled with high light output [17,18]. The material La_2_O_2_S provides an alternative to Gd_2_O_2_S for many scintillator-based applications. Compared with CaWO_4_—the conventional material used in X-ray intensifying screens—the sensitization factor of La_2_O_2_S:Tb for screen intensification is 3 to 5 times greater than CaWO_4_ [19], i.e., for the same X-Ray intensity input (and at the same energy) the La_2_O_2_S:Tb provides 3 to 5 times the intensity output. In addition, La_2_O_2_S:Eu has a high luminous efficiency and is widely used for X-ray luminescence measurements, light-storing, etc. In this investigation, the tested materials, from Phosphor Technology Ltd. (Herts, England), were CsI:Tl (DT81/VL-S1), Gd_2_O_2_S:Tb (UKL65/UF-R1), Gd_2_O_2_S:Pr (UKL59/UF-R1), La_2_O_2_S:Tb (SKL65/N-C1), and La_2_O_2_S:Eu (SKL63/F-R1).

**Figure 4:**
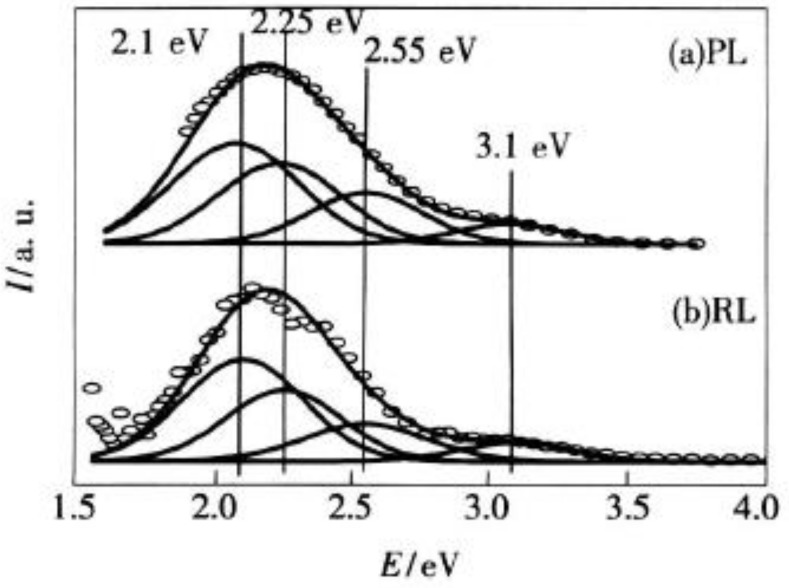
The spectrum of the CsI:Tl Scintillator with a Gaussian fitting calculation (**a**) the ultraviolet-exciting (310 nm) photoluminescence spectrum (PL) (**b**) the X-ray exciting radiation luminescence spectrum (RL) [20].

Wu Zhenglong [20] performed a Gaussian fit based calculation on the X-ray exciting radiation luminescence spectrum (RL) and the ultraviolet-exciting (310 nm) photoluminescence spectrum (PL) of CsI:Tl crystals (Figure 4).

The main peaks at 3.1, 2.55, 2.25, and 2.1 eV are emitted because of the electronic transition in *Tl^+^*, the $Tl^{+}-STE[Tl(I)-STE]$, and the $Tl_{2}^{++}-STE[Tl(II)-STE]$ [21,22].

**Figure 5:**
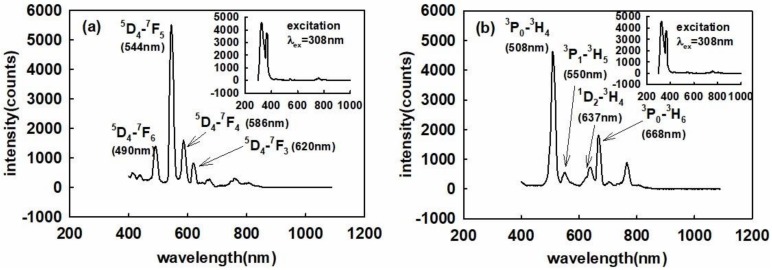
The emission spectra of the Gd_2_O_2_S (**a**) doped with Tb (**b**) doped with Pr.

Figure 5a shows the measured emission spectrum of Gd_2_O_2_S:Tb when used in the UVOFS configuration and under excitation using the 308 nm UV source, a UV fluorescence tube. It can be seen from Figure 5a that the emission spectrum consists of four main peaks located at wavelengths of 490, 544, 586, and 620 nm, the most intense peak being located at 544 nm. The four emission peaks of 490, 544, 586, and 620 nm correspond to the ^5^D_4_→^7^F_6_, ^5^D_4_→^7^F_5_, ^5^D_4_→^7^F_4_, and ^5^D_4_→^7^F_3_ transitions, respectively. The emission spectrum of Gd_2_O_2_S:Pr is shown in Figure 5b and was captured under the identical conditions as in the case of Gd_2_O_2_S:Tb. The spectrum of Figure 5b exhibits strong emission peaks from the energy transitions of ^3^P_0_→^3^H_4_ (508 nm), ^3^P_1_→^3^H_5_ (550 nm), ^1^D_2_→^3^H_4_ (637 nm) and ^3^P_0_→^3^H_6_ (668 nm) of Pr^3+^. The ^3^P_0_→^3^H_4_ (508 nm) is the dominant peak in the recorded spectrum.

**Figure 6:**
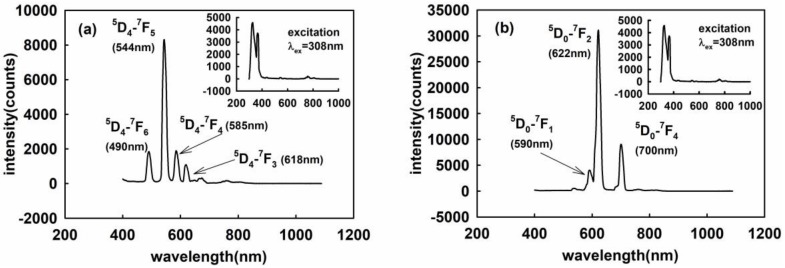
The emission spectra of the La_2_O_2_S (**a**) doped with Tb (**b**) doped with Eu.

Figure 6a,b show the fluorescence emission spectra of the Tb^3+^ and Eu^3+^ doped versions of La_2_O_2_S, respectively, when excitation was provided from the 308 nm UV source with the identical conditions as in the case of Figure 5. These emission peaks are attributed to the transitions ^5^D_4_→^7^F_J_ (*J* = 6–3) of Tb^3+^ and are indicated in Figure 6a, with the main peak located at 544 nm. It is clear that there are three emission peaks corresponding to the transitions ^5^D_0_→^7^F_1_ (590 nm), ^5^D_0_→^7^F_2_ (622 nm), and ^5^D_0_→^7^F_0_ (700 nm) from Eu^3+^ in Figure 6b. Therein, the intensity of the emission peak of ^5^D_0_→^7^F_2_ representing an electric dipole transition is the strongest, indicating that Eu^3+^ ions are located away from the inversion center.

## 3. Experimental Setup

**Figure 7:**
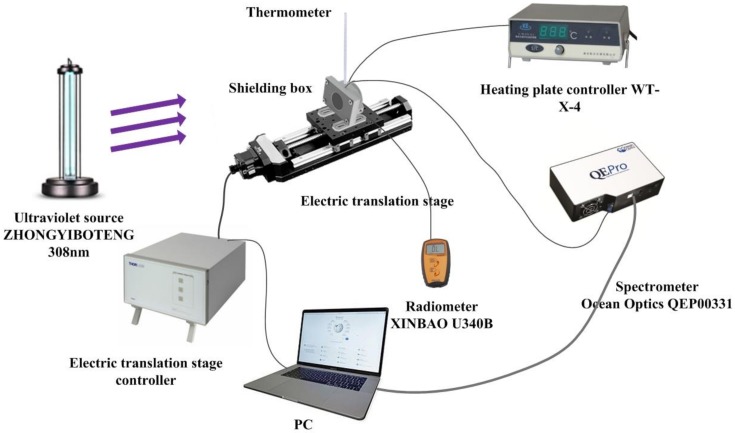
The experimental devices and method.

**Figure 8:**
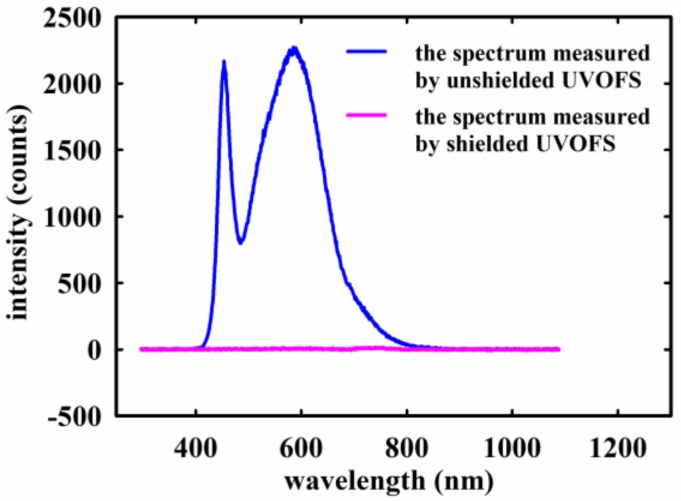
The comparison of the transmitted spectra from UVOFS without the shielding box and with the shielding box under visible light excitation.

The experimental set up is as shown in Figure 7. The UV source (ZHONGYIBOTENG UVB-308, Beijing, China) has a single output emission peak at 308 nm. It was necessary to operate (preheat) the UV source for approximately 30 min prior to use in the experiment to allow sufficient time for the output to stabilize. The shielding box was used in this experiment to reduce the possibility of interference from stray visible light from the UV source and the ambient environment. It is necessary to shield visible light, as it can be effectively transmitted through the probe of the sensor and the fiber and received as an extraneous noise signal by the spectrometer. The stray visible light transmitted can contaminate the fluorescence spectrum and this is clearly demonstrated in Figure 8. The shielding box was fixed on the electrically actuated translation stage, and the UV source was positioned such that the light output from it vertically illuminated the shielding box. The intensity of the incident UV radiation on the fiber sensor was simultaneously measured using a standard UV radiometer instrument (XINBAO U340B, Shenzhen, China) whose probe was located in close proximity with the UVOFS, and this provided a reference measurement of the light intensity incident from the UV source. The radiometer position was precisely adjusted by changing the distance between the shielding box and the UV source by moving the electrically actuated translation stage which allowed the incident UV intensity to be varied in a precise and repeatable manner. Simultaneously, the transmitted optical signal was detected at the distal end of the fiber sensor using the fluorescence Spectrometer (Ocean Optics QEPRO QEP00331). In this way, the linearity and repeatability of the optical fiber sensor could be accurately measured. The temperature characteristics of the sensor were also investigated using the arrangement shown in Figure 7 where the shielding box with a Mercury thermometer (resolution of 0.2 °C) was heated using a standard laboratory heating plate which allowed a maximum temperature of 400 °C to be tested.

In order to accurately measure the temporal resolution of the sensor, the spectrometer in Figure 7 was replaced with an MPPC (multi-pixel photon counter, HAMAMATSU MPPC C11208 series, Naka-ku Hamamatsu City, Japan) which is an instrument suitable for accurately measuring low-level fluorescence intensity as a function of time.

**Figure 9:**
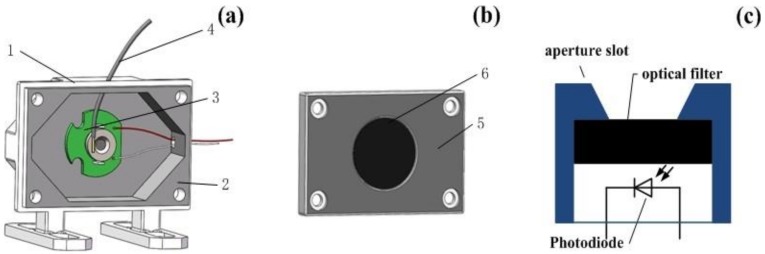
(**a**) The structure of the shielding box (**b**) The shielding cover (**c**) The structure of the UV radiometer (XINBAO U340B) with the OSRAM’s BPW66C photodiode, UVA + UVB detecting range, 2.5 times/second sampling rate and ±(4%FS + 2DGT) accuracy.

Figure 9 presents the structure of the shielding box. (1) is a box of ductile ABS (Acrylonitrile-Butadiene-Styrene) photosensitive resin; (2) is a film of PVC (Polyvinyl chloride); (3) is a UV radiometer (XINBAO U340B); (4) is the UVOFS, (5) is a shielding box cover; (6) is a UV transmission filter (ZWB2). It was necessary to apply a PVC film to the shielding box and its cover, due to the strong blue-violet light intensity generated from the UV source and concerns that the ABS photosensitive resin of the shielding box would not be capable of completely blocking the visible light. The cover plate of the shielding box (Figure 9b) was also fitted with a 3 cm filter diameter at its center. This provided necessary protection by filtering any visible optical signal above a wavelength of 400 nm. These components ensured that the UVOFS and radiometer received only the desired UV light signal from the source, eliminating the effects of visible light from the source and ambient noise.

## 4. Experimental Results

### 4.1. The Linearity of the Response to UV Light of the Scintillating Materials

**Figure 10:**
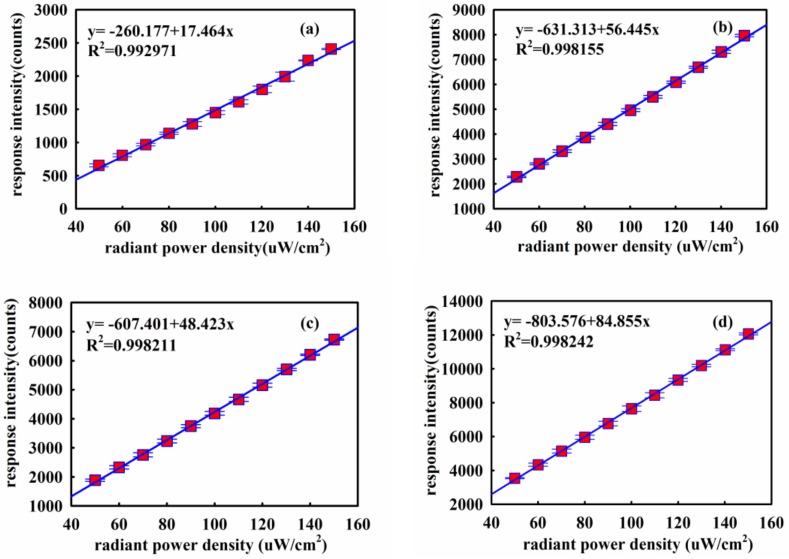

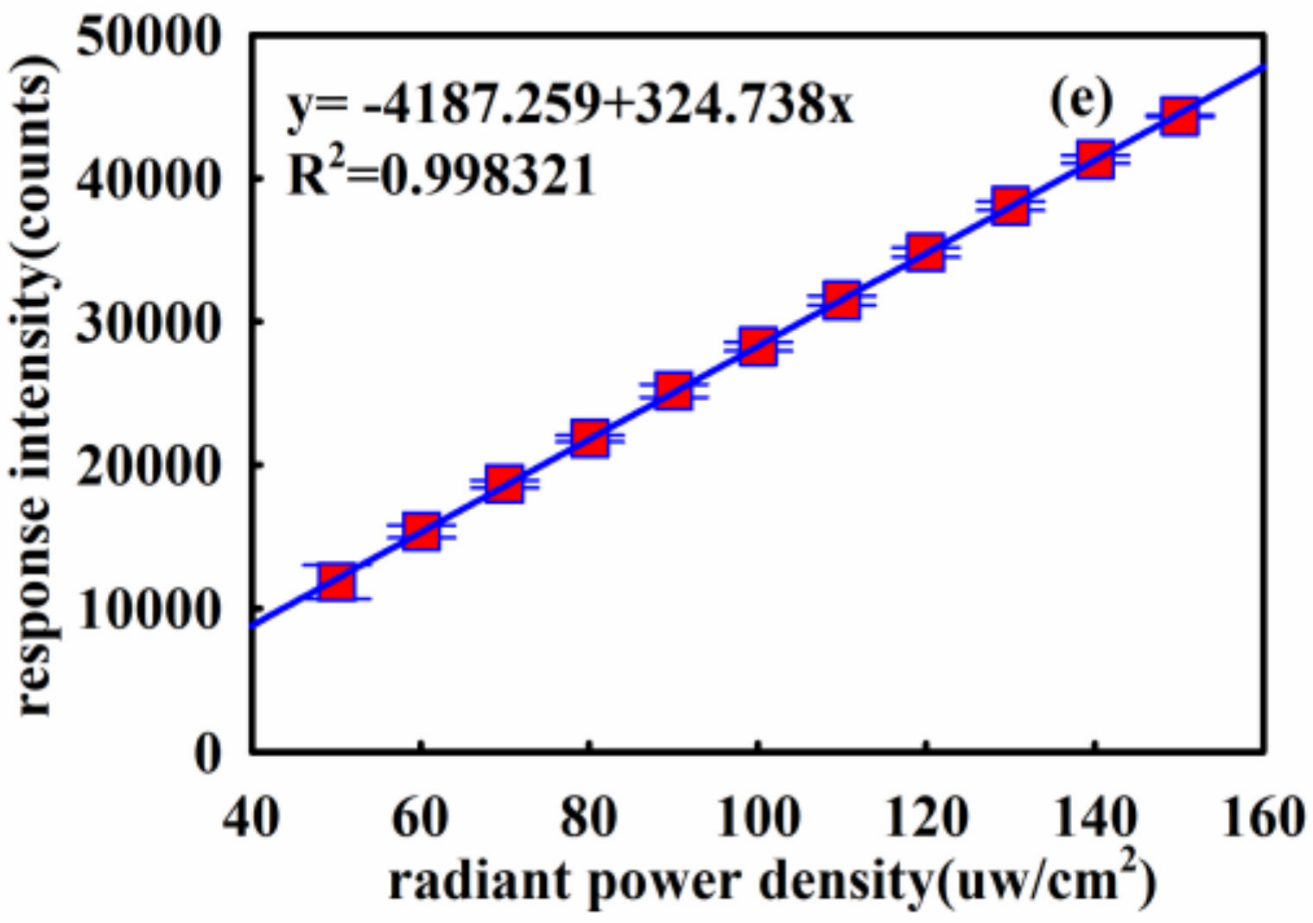
The measured relationship between the UV stimulation and response of the UVOFS based on the materials (**a**) CsI:Tl, (**b**) Gd_2_O_2_S:Tb, (**c**) Gd_2_O_2_S:Pr, (**d**) La_2_O_2_S:Tb, (**e**) La_2_O_2_S:Eu.

Five scintillating materials were tested as outlined in Section 3. The results from these tests at room temperature for the various materials for an incident radiant power density range from 50 to 150 μW/cm^2^ are shown in Figure 10.

The data points for response intensity in the diagram were derived from the maximum value of the main peak of the emission of the materials (as indicated in Figure 3). It is clear that the UVOFS based on these five materials exhibits a good linear response in each case with *R*^2^ > 0.99. Error bars representing the output of an error analysis are included in the plots of Figure 10a–e but were so small that they are masked by the symbol representing the data point in each case. All the fitting lines have a negative offset because the radiometer (a semiconductor device) has a positive offset when it receives UV light. The UVOFS based on the La_2_O_2_S:Eu scintillating material exhibited the highest coefficient of determination (*R*^2^ = 0.998) and the highest response magnitude for the same irradiation intensity. The results of this section indicate that the UVOFS based on the La_2_O_2_S:Eu scintillator is, therefore, the best candidate for UV measurement. La_2_O_2_S:Eu was subsequently selected for the rest of the characterization of the UVOFS.

Table 1 shows the error analysis of the La_2_O_2_S:Eu-based UVOFS using the fitting function calculation and this indicates that the error of all points except the first point is less than 2%. Actually, the error of the sensor is less than 2%, which will be detailed further in Section 4.4.

### 4.2. Repeatability of the UVOFS

**Figure 11:**
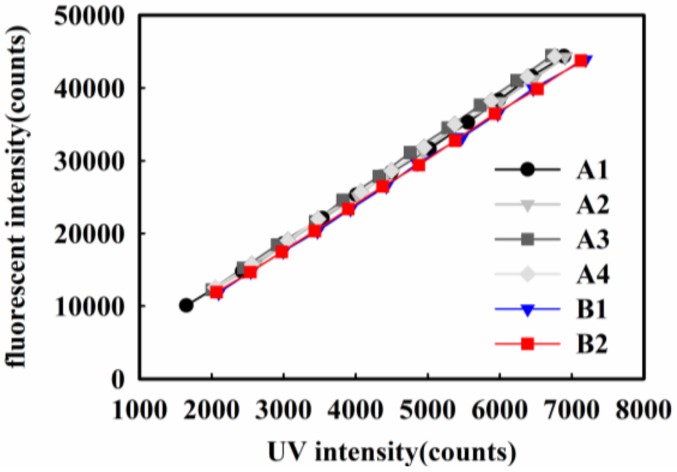
The response of the output intensity of UVOFS versus UV stimulation repeated in 6 cycles to establish the repeatability of the sensor.

Two sets of experiments were performed over an interval of 4 days. The first experiment comprised of four cycles of identical repeated tests and marked as A1 to A4 in Figure 11. The second experiment was undertaken after 4 days to test the repeatability and the results were marked as B1 and B2 in Figure 11. The UV intensity was measured as the maximum value (the value of the main peak) of the UV light based on the spectra as shown in Figure 3. The results of Figure 11 show the repeatability in the repeated data set. However, the small differences that exist between the two sets of data (captured on different days) can be attributed to some very small but unavoidable movements in position of the sensor relative to the radiometer in the time that elapsed between the two experiments. The unavoidable movement came from the slight deformation of the blu-tack (Bostik, Thomastown, Australia) for fixing the UVOFS probe on the surface of UV radiometer.

### 4.3. UVOFS Temperature Characteristics

**Figure 12:**
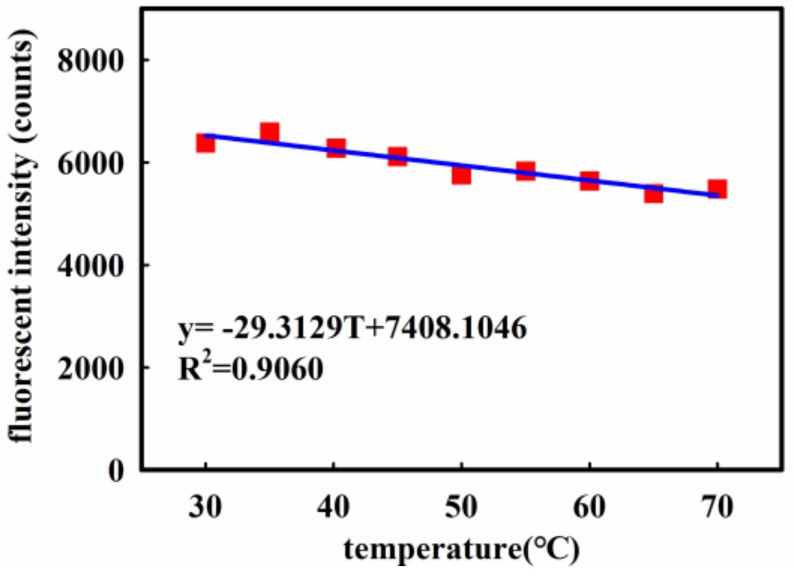
The response of the UVOFS versus temperature.

To test the temperature response of the UVOFS, the ambient temperature was changed by adjusting the heating device (hot plate) fixed to the shielding box. This allowed a temperature range of 30 to 70 °C to be achieved. Measurements were made at incremental steps of 5 °C. The test results are shown in Figure 12.

A linear regression analysis was applied to the data of Figure 12 (*R*^2^ = 0.9060) and the following relationship was established which describes the temperature response (approximated to a linear function):

(1) y=−29.3129T+7408.1046 

With the linear fitting curve at room temperature in Figure 10e, the following expression can be used:

(2) $$\Delta x=\frac{-29.3129\cdot \Delta T}{324.738}\;$$

where y is the response intensity (counts), T is the temperature (°C), Δx is a changing radiant power density value (μW/cm^2^) measured using the radiometer in the presence of changing temperature.

If the temperature influence of the sensor is not taken into account when measuring, and the ambient temperature changes by 10 °C, that is ΔT = 10 °C, the error in the sensor signal is equivalent to a radiant flux value of 0.90 μW/cm^2^. The value of the temperature coefficient of the sensor is so small that the temperature effects can be ignored in the circumstance where the accuracy of the request is not very high.

### 4.4. UVOFS Response Time Analysis

**Figure 13:**
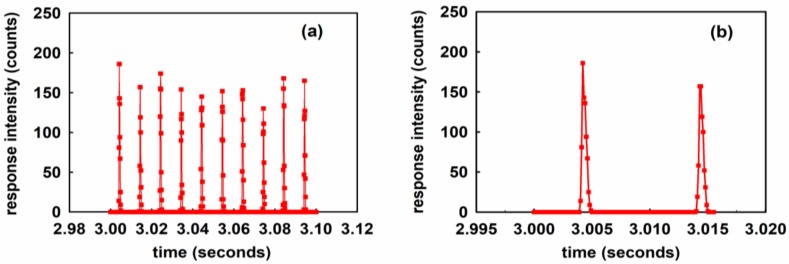
The time-resolved output signals from the UVOFS captured using the MPPC detector with the gate time set to 0.1 ms (**a**) within 0.1 s; (**b**) within 0.015 s.

The response time of the sensor is very important, especially in the case of the La_2_O_2_S:Eu scintillator, as it is often used as a ‘long afterglow’ material. If the afterglow decay time is too long, light signals excited by successive UV light pulses will be superimposed so that the UV radiation intensity cannot be accurately measured in real time. The measurement of the time response was performed, using the same system set up as described in Section 3, but the spectrometer was replaced by the MPPC (HAMAMATSU MPPC C11208 series) device. For this, the gate time (the time for which the photodiode array records the incoming light signal) was set to 100 μs, which corresponds to a sampling frequency of approximately 1 × 10^4^ Hz. The experimental results are shown in Figure 13.

Figure 13a shows the experimental data collected during a 0.1 s time interval after the output power of the UV source was stabilized. It can be seen from Figure 13 that the MPPC collected 10 pulses in the 0.1 s interval, (corresponding to 100 pulses during in a 1 s interval). China’s urban AC power supply frequency is 50 Hz, which means that the UV source, a fluorescent tube, flashes 100 times per second, which is consistent with the result of Figure 13a, which indicates that the pulse signals collected in Figure 13a are the real UV light pulses emitted by the UV source. Figure 13b includes a limited time interval of 15.5 ms (corresponding to the ‘absolute’ time values of 3.0000 s~3.0155 s in Figure 13a) showing the ‘zoomed in’ highly temporally resolved signals captured during this time. The relatively high sampling frequency capability of the MPPC captured 151 data points during this time, which is enough to accurately capture the detail of the pulse signal. Each of the measured UV source output pulse widths is less than 1 ms in Figure 13b, which means that the sampling frequency of the fiber optic sensor is at worst 1000 Hz. This is much higher than the sampling frequency of the semiconductor detectors that are currently commercially available. The UVOFS of this investigation is therefore capable of accurately measuring and faithfully reproducing (in real time) the output pulses of the UV source used in this investigation.

Table 2 shows 8 independent sets of data collected at two radiant power density values of 50 and 60 μW/cm^2^ as measured on the reference radiometer instrument. The UV intensity was recorded using the UVOFS and spectrometer configuration as described in Figure 7 and the resulting Spectrum of Figure 3. It can be seen that the UV intensity (counts) is consistent with the trend of the fluorescence intensity (counts) from the UVOFS. However, there are some different trends in the evidence between UV intensity (counts) and the value from the radiometer (μW/cm^2^). From Figure 13 it was determined that the bandwidth of the UVOFS is at least 1 kHz, while the sampling frequency of the radiometer is only 2.5 Hz (as from the manufacturer’s data). Therefore, if the UV source has some small and short-lived fluctuation in intensity, e.g., transient power surges in the power supply, the UVOFS can capture it but the radiometer cannot. That is the major advantage of the UVOFS system described in this article.

### 4.5. Comparison of the UVOFS and an Ultraviolet Radiometer

**Figure 14:**
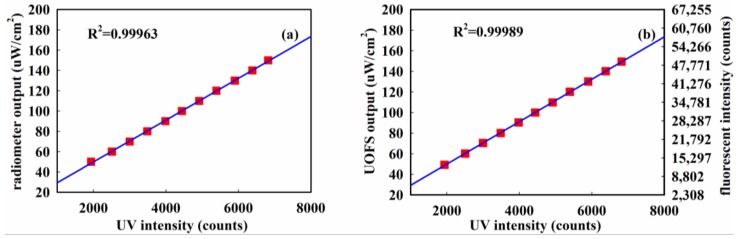
The comparison of the (**a**) ultraviolet radiometer and (**b**) UVOFS.

Based on the datasheet of the spectrometer, the output of the UVOFS and UV radiometer could be further compared as shown in Figure 14.

All data points in Figure 14 are from the average of four sets of data detected by the UVOFS and the radiometer. It can be seen that the determination coefficient of the UVOFS is slightly larger than the UV radiometer, showing that the linear response of the UVOFS is superior when compared to the radiometer instrument. Therefore, the comparison provides further evidence to prove that the UVFOS is capable of accurately detecting UV radiation.

## 5. Conclusions

A novel ultraviolet optical fiber sensor based on a La_2_O_2_S:Eu scintillator has been designed, tested, and compared with a commercially available UV detector instrument. The principle of operation of the optical fiber sensor with the scintillating materials was introduced, and characteristic features such as the linear response to UV radiation intensity, reproducibility, temperature response, time response (sampling frequency) has been presented. The experimental results show that this novel system has excellent linearity (*R*^2^ = 0.998) and repeatability, a small temperature coefficient (9.03 × 10^−2^ (μW/cm^2^)/°C) as well as a high sampling frequency capability (at least 1000 Hz) under UV irradiation at 308 nm. With such a small temperature coefficient, there is no need to consider the temperature effects for most applications, as a 10 °C variation represents only 0.90 μW/cm^2^ change in the radiant power density of the input light. The novel structure is capable of detecting minor amplitude fluctuations, even pulsed UV light waveforms in real-time, which is not a feature afforded by the UV radiometer instrument. In addition, the sensor structure of this investigation is immune to external electromagnetic interference, so it could be a good candidate to detect UV light in hostile electrical environments.

## Figures and Tables

**Table 1:** The error analysis of the La_2_O_2_S:Eu-based UVOFS.

Radiometer (μW/cm^2^)	UVOFS (μW/cm^2^)	Error (%)
50	51.33	2.27
60	60.78	1.30
70	70.40	0.58
80	80.13	0.16
90	90.39	0.43
100	99.95	0.05
110	109.79	0.20
120	120.20	0.17
130	130.18	0.14
140	140.17	0.12
150	149.47	0.35

**Table 2:** The response from radiometer and UVOFS under radiant power density values of 50 and 60 μW/cm^2^.

Test Group	Radiometer (μW/cm^2^)	UV Intensity (counts)	Fluorescence Intensity (counts)	UVOFS (μW/cm^2^)
1	50.0	1649	10,072	43.91
2	50.0	2037	12,388	51.04
3	50.2	2002	12,288	50.73
4	50.0	2052	12,579	51.63
5	60.0	2425	14,783	58.42
6	60.2	2604	15,643	61.07
7	60.0	2442	15,264	59.90
8	60.0	2560	15,744	61.38
